# Supplementary material for: Functional analysis within latent states: A novel framework for analysing functional time series data
Source: PLoS One. 2025 Jun 27;20(6):e0326598. doi: 10.1371/journal.pone.0326598 (PMC12204692; doi:10.1371/journal.pone.0326598)
Supplement: Supplementary file 1 — **S1 File. Supplementary Materials** Supplementary materials for manuscript: “Functional analysis within latent states: A novel framework for analysing functional time series data” (PDF) [file pone.0326598.s001.pdf]

Supplementary Materials

Supplementary materials for manuscript: ”Functional analysis within latent states: A novel framework for analysing functional time series data”

1.1 Initialisation of FHMM with Centroids from Subsampled Models

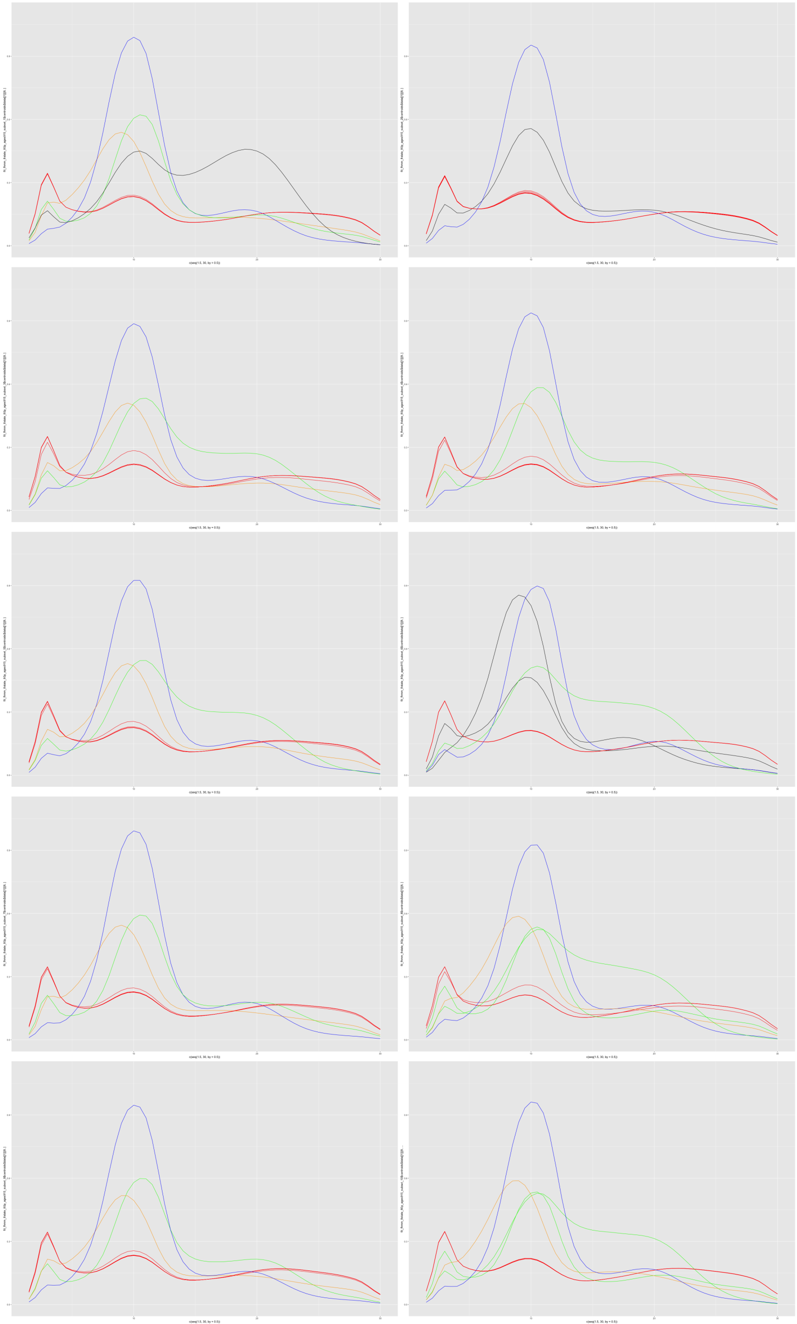

Supplementary Fig 1: Functional latent state centroids calculated from 10 subsampled models, each fit with 8 latent states using an 80% random subsample of the total dataset.

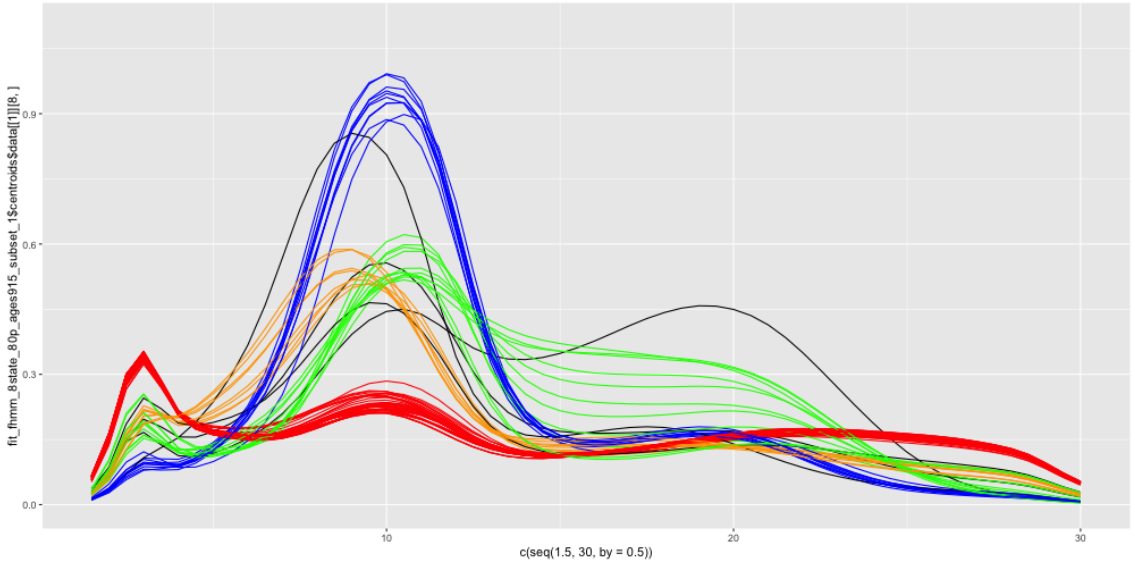

Supplementary Fig 2: Functional latent state centroids from 10 subsampled models plotted together, with heuristically grouped sets of similar centroids in matched colours.

1.2 ANOVA Results Between Dominant Latent States

Supplementary Table 1: ANOVA Results for Age, Sex and EHQ (Edinburgh Handedness Questionnaire) between individuals spending 80% or more of their time in one functional latent state

ANOVA	Df	Sum Sq	Mean Sq	F value	$Pr(> F)$
Age	1	29.8	29.762	10.91	0.00108
Sex	1	0.02	0.02251	0.101	0.751
EHQ	1	4158	4158	1.691	0.195

1.3 FPCA Scree Plots

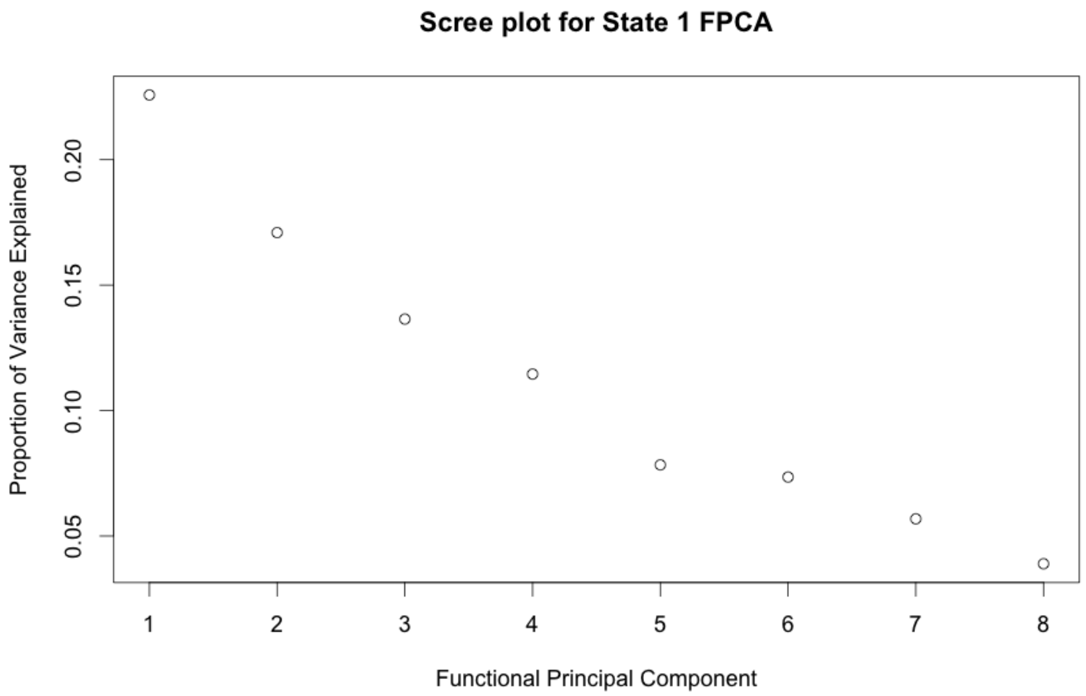

Supplementary Fig 3: Scree plot for FPCA in State 1

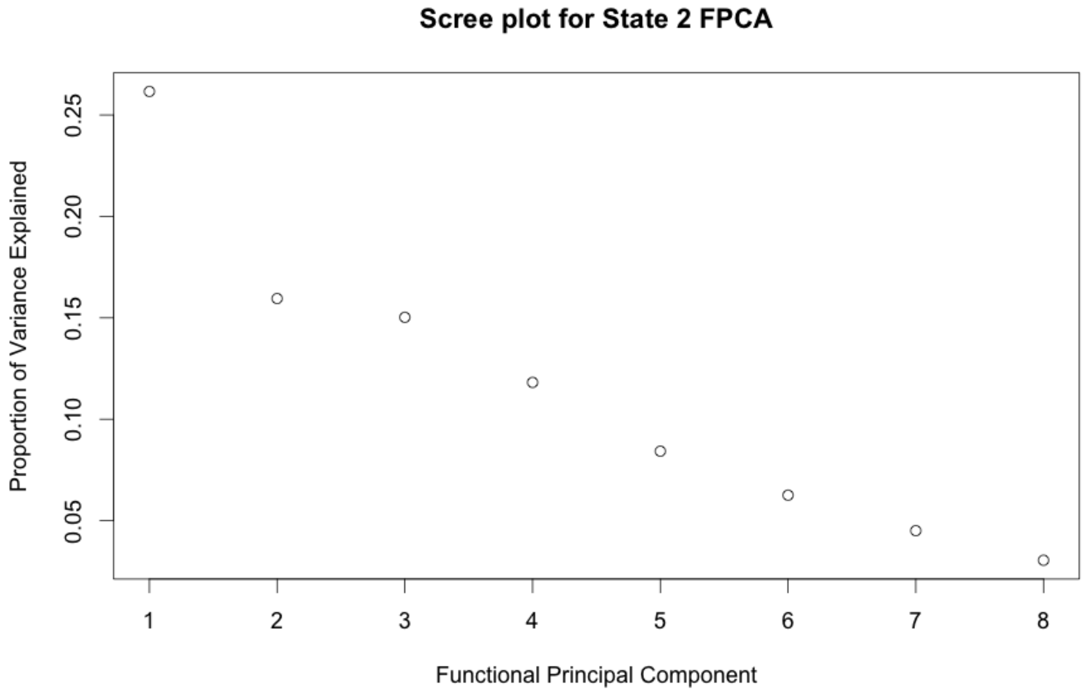

Supplementary Fig 4: Scree plot for FPCA in State 2

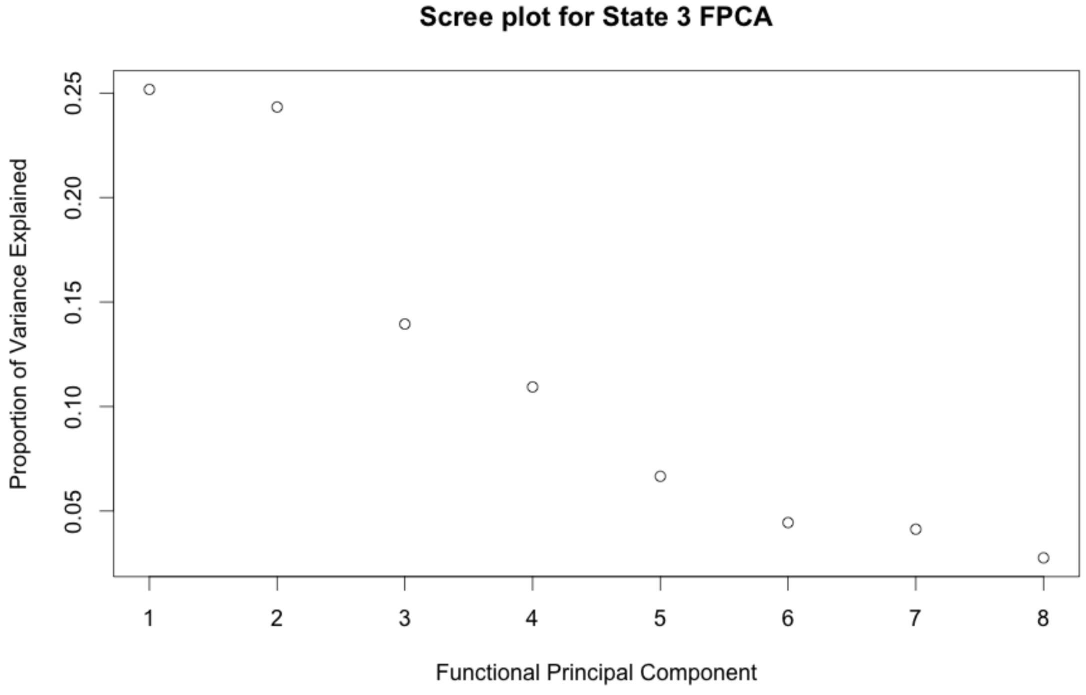

Supplementary Fig 5: Scree plot for FPCA in State 3

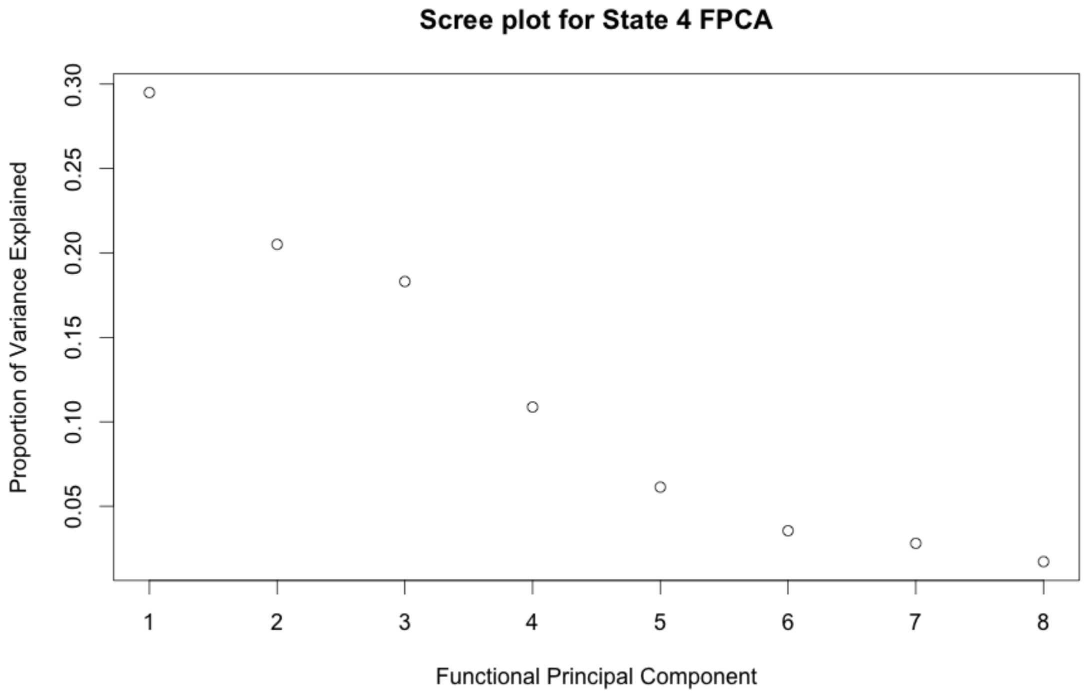

Supplementary Fig 6: Scree plot for FPCA in State 4

1.4 Functional Principal Components for State 2

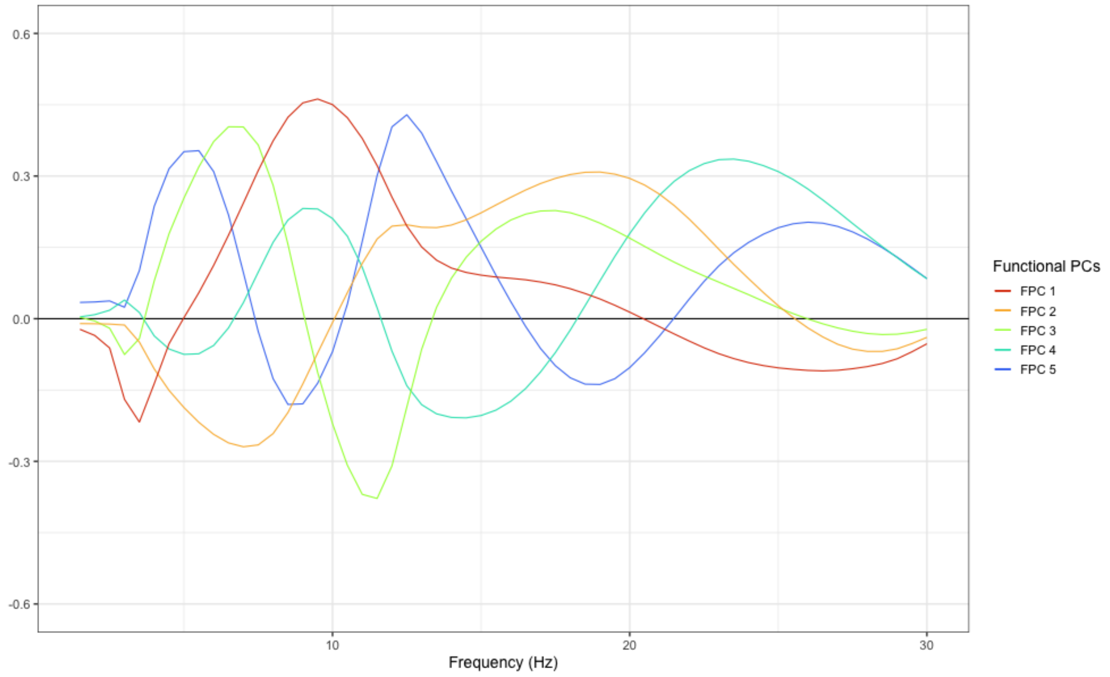

Supplementary Fig 7: Functional principal components (eigenfunctions) for observations allocated to state 2.

1.5 Functional Principal Components for State 4

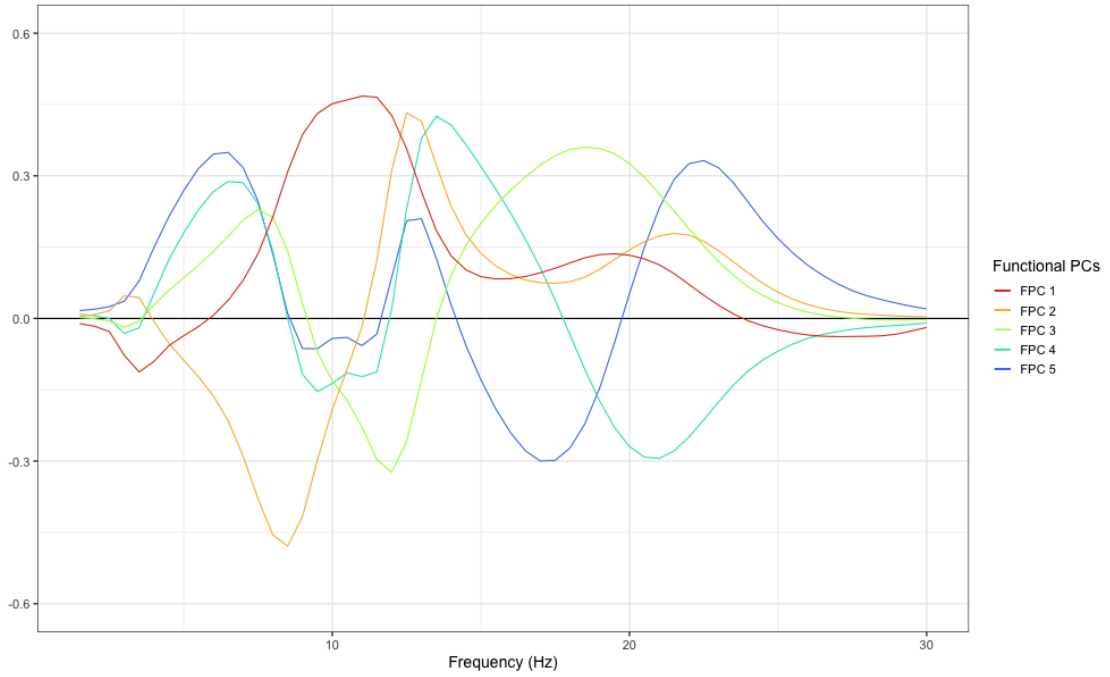

Supplementary Fig 8: Functional principal components (eigenfunctions) for observations allocated to state 4.

1.6 Full Bayesian Regression Model Table for State 1

Supplementary Table 2: Regression coefficients and 95% credible intervals from Bayesian regression model for state 1.

Independent Variable	Regression Coefficient	95% Credible Interval	Dependent Variable
Intercept			
Intercept	-11	-52, 31	MFQ SR
Intercept	-17	-75, 41	SCARED SR
Intercept	20	-24, 64	YSR Ext
Intercept	32	-13, 78	YSR Int
Intercept	115	46, 184	NIH Card
Intercept	93	39, 146	NIH Flanker
Intercept	131	72, 191	NIH List
Intercept	42	-46, 128	NIH Pattern
Sex = Female			
Sex = Female	2.6	-1.8, 7.0	MFQ SR
Sex = Female	5.6	-0.69, 12	SCARED SR
Sex = Female	-0.66	-5.3, 3.9	YSR Ext
Sex = Female	-1.9	-6.8, 2.9	YSR Int
Sex = Female	-2.7	-10, 4.5	NIH Card
Sex = Female	-3.5	-9.3, 2.1	NIH Flanker
Sex = Female	-3.1	-9.6, 3.3	NIH List
Sex = Female	4.5	-4.5, 14	NIH Pattern
Age			
Age	0.34	-1.6, 2.3	MFQ SR
Age	-0.52	-3.3, 2.3	SCARED SR
Age	1.1	-0.95, 3.2	YSR Ext
Age	0.11	-2.0, 2.3	YSR Int
Age	0.18	-3.1, 3.4	NIH Card
Age	-0.93	-3.4, 1.6	NIH Flanker
Age	0.82	-2.0, 3.7	NIH List
Age	4.4	0.33, 8.4	NIH Pattern
EHQ			
EHQ	0.03	-0.03, 0.08	MFQ SR
EHQ	0.01	-0.07, 0.08	SCARED SR
EHQ	0.05	0.00, 0.11	YSR Ext
EHQ	0.03	-0.02, 0.09	YSR Int
EHQ	-0.02	-0.11, 0.07	NIH Card
EHQ	-0.01	-0.07, 0.06	NIH Flanker
EHQ	-0.07	-0.15, 0.00	NIH List
EHQ	-0.02	-0.13, 0.08	NIH Pattern
N. States			
N. States	1.7	-6.2, 9.5	MFQ SR
N. States	8	-3.3, 19	SCARED SR
N. States	3.7	-4.3, 12	YSR Ext
N. States	2.4	-6.1, 11	YSR Int
N. States	-6.2	-19, 6.5	NIH Card
N. States	-9.2	-20, 0.81	NIH Flanker
N. States	-8.7	-20, 3.0	NIH List
N. States	4.6	-12, 21	NIH Pattern
N. Transitions			
N. Transitions	0.51	-2.5, 3.6	MFQ SR
N. Transitions	-1.1	-5.4, 3.2	SCARED SR
N. Transitions	-0.78	-3.9, 2.4	YSR Ext

N. Transitions	0.33	-3.0, 3.7	YSR Int
N. Transitions	3.2	-1.8, 8.2	NIH Card
N. Transitions	6	2.0, 9.9	NIH Flanker
N. Transitions	2.3	-2.2, 6.7	NIH List
N. Transitions	-1.1	-7.4, 5.2	NIH Pattern
Dominant State %			
Dominant State %	14	-14, 43	MFQ SR
Dominant State %	35	-5.1, 75	SCARED SR
Dominant State %	13	-17, 42	YSR Ext
Dominant State %	21	-11, 51	YSR Int
Dominant State %	-19	-65, 28	NIH Card
Dominant State %	14	-23, 50	NIH Flanker
Dominant State %	-29	-70, 11	NIH List
Dominant State %	-11	-71, 48	NIH Pattern
S1 FPC1			
S1 FPC1	-4.9	-19, 9.6	MFQ SR
S1 FPC1	-4.2	-25, 16	SCARED SR
S1 FPC1	-4.2	-19, 11	YSR Ext
S1 FPC1	-9.7	-26, 6.0	YSR Int
S1 FPC1	-13	-36, 10	NIH Card
S1 FPC1	-12	-30, 7.0	NIH Flanker
S1 FPC1	3	-18, 24	NIH List
S1 FPC1	-9.6	-39, 20	NIH Pattern
S1 FPC2			
S1 FPC2	3.3	-9.3, 16	MFQ SR
S1 FPC2	16	-1.7, 34	SCARED SR
S1 FPC2	-0.35	-13, 12	YSR Ext
S1 FPC2	1.7	-12, 15	YSR Int
S1 FPC2	-13	-34, 7.4	NIH Card
S1 FPC2	7.4	-8.6, 23	NIH Flanker
S1 FPC2	-10	-28, 8.1	NIH List
S1 FPC2	4.2	-21, 30	NIH Pattern
S1 FPC3			
S1 FPC3	-7.7	-34, 19	MFQ SR
S1 FPC3	-3.8	-42, 34	SCARED SR
S1 FPC3	5.6	-22, 33	YSR Ext
S1 FPC3	-6.2	-35, 23	YSR Int
S1 FPC3	-32	-76, 11	NIH Card
S1 FPC3	-16	-49, 18	NIH Flanker
S1 FPC3	-33	-71, 5.9	NIH List
S1 FPC3	-59	-113, -4.6	NIH Pattern
S1 FPC4			
S1 FPC4	1.7	-14, 18	MFQ SR
S1 FPC4	-7.6	-30, 14	SCARED SR
S1 FPC4	4.2	-12, 20	YSR Ext
S1 FPC4	3.6	-13, 21	YSR Int
S1 FPC4	-31	-57, -5.4	NIH Card
S1 FPC4	-25	-45, -4.7	NIH Flanker
S1 FPC4	-25	-48, -2.2	NIH List
S1 FPC4	-23	-56, 9.4	NIH Pattern
S1 FPC5			
S1 FPC5	0.9	-33, 35	MFQ SR
S1 FPC5	-30	-78, 19	SCARED SR
S1 FPC5	17	-19, 53	YSR Ext
S1 FPC5	-0.73	-38, 37	YSR Int
S1 FPC5	8.3	-47, 65	NIH Card

S1 FPC5	5.3	-38, 49	NIH Flanker
S1 FPC5	-21	-70, 29	NIH List
S1 FPC5	-59	-129, 11	NIH Pattern

Supplementary Table 2: EHQ = Edinburgh Handedness Questionnaire; S1 FPC1 = Functional principal component 1 for state 1; MFQ SR = Mood and Feelings Questionnaire, Self Report; SCARED SR = Screen for Child Anxiety Related Disorders, Self Report; YSR Ext = Youth Self Report, Externalising Scale; YSR Int = Youth Self Report, Internalising Scale; NIH = National Institutes of Health Toolbox Cognitive function tasks; NIH Card = Card Sorting task measuring executive function; NIH Flanker = Flanker task measuring executive function and attention; NIH List = List sorting task measuring working memory; NIH Pattern = Pattern comparison task measuring processing speed.

1.7 Full Bayesian Regression Model Table for State 2

Supplementary Table 3: Regression coefficients and 95% credible intervals from Bayesian regression model for state 2.

Independent Variable	Regression Coefficient	95% Credible Interval	Dependent Variable
(Intercept)			
(Intercept)	-21	-71, 29	MFQ SR
(Intercept)	-26	-101, 50	SCARED SR
(Intercept)	22	-36, 80	YSR Ext
(Intercept)	27	-40, 94	YSR Int
(Intercept)	77	2.4, 152	NIH Card
(Intercept)	138	66, 210	NIH Flanker
(Intercept)	60	-34, 153	NIH List
(Intercept)	36	-92, 161	NIH Pattern
Sex = Female	6.2	-1.0, 14	MFQ SR
Sex = Female	11	0.67, 22	SCARED SR
Sex = Female	3.8	-4.6, 12	YSR Ext
Sex = Female	0.75	-9.0, 10	YSR Int
Sex = Female	6.5	-4.2, 17	NIH Card
Sex = Female	1.1	-9.2, 11	NIH Flanker
Sex = Female	4.7	-8.4, 18	NIH List
Sex = Female	-1.6	-20, 16	NIH Pattern
Age	-0.15	-2.2, 1.9	MFQ SR
Age	0.21	-3.0, 3.3	SCARED SR
Age	-0.6	-3.0, 1.8	YSR Ext
Age	-0.9	-3.7, 1.9	YSR Int
Age	2	-1.2, 5.0	NIH Card
Age	-1.6	-4.5, 1.4	NIH Flanker
Age	1.9	-2.0, 5.8	NIH List
Age	7.6	2.3, 13	NIH Pattern
EHQ	0.01	-0.08, 0.10	MFQ SR
EHQ	0.06	-0.08, 0.19	SCARED SR
EHQ	0.01	-0.10, 0.11	YSR Ext
EHQ	0.01	-0.10, 0.13	YSR Int
EHQ	-0.02	-0.16, 0.11	NIH Card
EHQ	0.02	-0.11, 0.14	NIH Flanker
EHQ	0.12	-0.04, 0.29	NIH List
EHQ	0.02	-0.20, 0.24	NIH Pattern
N. States	3.3	-3.6, 10	MFQ SR
N. States	7.1	-3.2, 18	SCARED SR
N. States	2.3	-5.7, 10	YSR Ext
N. States	4.7	-4.5, 14	YSR Int
N. States	-2.2	-12, 8.0	NIH Card
N. States	-2.2	-12, 7.6	NIH Flanker
N. States	-2.9	-15, 9.8	NIH List
N. States	-2.4	-19, 15	NIH Pattern
N. Transitions	-0.32	-4.6, 3.9	MFQ SR
N. Transitions	-3.1	-9.3, 3.2	SCARED SR
N. Transitions	1.3	-3.5, 6.1	YSR Ext
N. Transitions	-1.2	-6.8, 4.3	YSR Int
N. Transitions	-2.1	-8.3, 4.0	NIH Card
N. Transitions	-2.4	-8.3, 3.5	NIH Flanker
N. Transitions	2.1	-5.4, 9.8	NIH List
N. Transitions	-2.2	-12, 8.2	NIH Pattern

Dominant State %	31	-5.5, 68	MFQ SR
Dominant State %	34	-20, 88	SCARED SR
Dominant State %	34	-7.4, 76	YSR Ext
Dominant State %	38	-10, 86	YSR Int
Dominant State %	-0.9	-55, 53	NIH Card
Dominant State %	-32	-83, 20	NIH Flanker
Dominant State %	9.9	-57, 78	NIH List
Dominant State %	-26	-117, 67	NIH Pattern
S2 FPC1	-4.5	-19, 9.9	MFQ SR
S2 FPC1	-7.8	-29, 14	SCARED SR
S2 FPC1	4.4	-12, 21	YSR Ext
S2 FPC1	-5	-24, 14	YSR Int
S2 FPC1	12	-9.1, 33	NIH Card
S2 FPC1	14	-6.0, 34	NIH Flanker
S2 FPC1	-5.8	-32, 21	NIH List
S2 FPC1	9.8	-25, 45	NIH Pattern
S2 FPC2	6.3	-10, 23	MFQ SR
S2 FPC2	13	-12, 38	SCARED SR
S2 FPC2	-8.6	-28, 11	YSR Ext
S2 FPC2	3.9	-18, 26	YSR Int
S2 FPC2	9.7	-14, 34	NIH Card
S2 FPC2	0.78	-22, 24	NIH Flanker
S2 FPC2	6	-24, 37	NIH List
S2 FPC2	26	-15, 66	NIH Pattern
S2 FPC3	4.3	-9.0, 17	MFQ SR
S2 FPC3	-0.7	-21, 19	SCARED SR
S2 FPC3	2.7	-13, 18	YSR Ext
S2 FPC3	3.6	-14, 21	YSR Int
S2 FPC3	-20	-40, -1.2	NIH Card
S2 FPC3	-11	-30, 7.4	NIH Flanker
S2 FPC3	-5.2	-29, 19	NIH List
S2 FPC3	-30	-62, 3.1	NIH Pattern
S2 FPC4	24	-2.8, 51	MFQ SR
S2 FPC4	16	-24, 57	SCARED SR
S2 FPC4	19	-12, 50	YSR Ext
S2 FPC4	28	-7.5, 64	YSR Int
S2 FPC4	-2.1	-42, 38	NIH Card
S2 FPC4	-28	-66, 10	NIH Flanker
S2 FPC4	-33	-83, 17	NIH List
S2 FPC4	-70	-137, -3.5	NIH Pattern
S2 FPC5	30	1.9, 58	MFQ SR
S2 FPC5	46	4.0, 88	SCARED SR
S2 FPC5	19	-14, 52	YSR Ext
S2 FPC5	24	-14, 61	YSR Int
S2 FPC5	52	11, 94	NIH Card
S2 FPC5	-8	-48, 31	NIH Flanker
S2 FPC5	17	-35, 68	NIH List
S2 FPC5	37	-34, 107	NIH Pattern

Supplementary Table 3: EHQ = Edinburgh Handedness Questionnaire; S2 FPC1 = score on functional principal component 1 for state 2; MFQ SR = Mood and Feelings Questionnaire, Self Report; SCARED SR = Screen for Child Anxiety Related Disorders, Self Report; YSR Ext = Youth Self Report, Externalising Scale; YSR Int = Youth Self Report, Internalising Scale; NIH = National Institutes of Health Toolbox Cognitive function tasks; NIH Card = Card Sorting task measuring executive function; NIH Flanker = Flanker task measuring executive function and attention; NIH List = List sorting task measuring working memory; NIH Pattern = Pattern comparison task measuring processing speed.

1.8 Full Bayesian Regression Model Table for State 3

Supplementary Table 4: Regression coefficients and 95% credible intervals from Bayesian regression model for state 3.

Independent Variable	Regression Coefficient	95% Credible Interval	Dependent Variable
Intercept			
Intercept	20	-33, 71	MFQ SR
Intercept	85	12, 158	SCARED SR
Intercept	47	-6.3, 99	YSR Ext
Intercept	93	38, 148	YSR Int
Intercept	158	56, 262	NIH Card
Intercept	116	49, 183	NIH Flanker
Intercept	112	4.4, 218	NIH List
Intercept	-55	-195, 85	NIH Pattern
Sex = Female			
Sex = Female	5.4	-1.6, 12	MFQ SR
Sex = Female	3.8	-5.9, 13	SCARED SR
Sex = Female	2.9	-4.1, 10	YSR Ext
Sex = Female	-0.87	-8.3, 6.4	YSR Int
Sex = Female	-3	-17, 11	NIH Card
Sex = Female	-6.5	-15, 2.5	NIH Flanker
Sex = Female	-0.72	-15, 13	NIH List
Sex = Female	17	-1.2, 36	NIH Pattern
Age			
Age	0.09	-2.6, 2.8	MFQ SR
Age	-2.4	-6.1, 1.2	SCARED SR
Age	0.02	-2.7, 2.7	YSR Ext
Age	-0.71	-3.5, 2.1	YSR Int
Age	-0.49	-5.8, 4.8	NIH Card
Age	0.39	-3.1, 3.8	NIH Flanker
Age	-0.1	-5.6, 5.4	NIH List
Age	3.7	-3.4, 11	NIH Pattern
EHQ			
EHQ	-0.04	-0.10, 0.01	MFQ SR
EHQ	-0.02	-0.10, 0.06	SCARED SR
EHQ	-0.01	-0.07, 0.04	YSR Ext
EHQ	-0.02	-0.08, 0.04	YSR Int
EHQ	-0.08	-0.19, 0.03	NIH Card
EHQ	0	-0.07, 0.07	NIH Flanker
EHQ	0.06	-0.06, 0.17	NIH List
EHQ	-0.09	-0.24, 0.05	NIH Pattern
N. States			
N. States	4.1	-3.1, 11	MFQ SR
N. States	3.5	-6.5, 14	SCARED SR
N. States	7.3	0.11, 15	YSR Ext
N. States	2.4	-5.1, 9.8	YSR Int
N. States	-6.5	-21, 7.6	NIH Card
N. States	-3	-12, 6.2	NIH Flanker
N. States	1.8	-13, 16	NIH List
N. States	9.1	-9.8, 28	NIH Pattern
N. Transitions			
N. Transitions	-2.8	-5.8, 0.27	MFQ SR
N. Transitions	-3.9	-8.1, 0.34	SCARED SR
N. Transitions	-2.5	-5.5, 0.45	YSR Ext

N. Transitions	-4.5	-7.7, -1.3	YSR Int
N. Transitions	-2.9	-8.9, 3.1	NIH Card
N. Transitions	-1.9	-5.8, 2.1	NIH Flanker
N. Transitions	-2	-8.2, 4.2	NIH List
N. Transitions	3.3	-4.6, 11	NIH Pattern
Dominant State %			
Dominant State %	-16	-47, 16	MFQ SR
Dominant State %	-45	-89, -1.1	SCARED SR
Dominant State %	-7.2	-38, 24	YSR Ext
Dominant State %	-33	-66, 0.03	YSR Int
Dominant State %	-42	-105, 19	NIH Card
Dominant State %	-30	-70, 11	NIH Flanker
Dominant State %	-17	-81, 47	NIH List
Dominant State %	101	15, 185	NIH Pattern
S3 FPC1			
S3 FPC1	1.4	-14, 17	MFQ SR
S3 FPC1	8.4	-13, 30	SCARED SR
S3 FPC1	1	-14, 16	YSR Ext
S3 FPC1	0.23	-16, 16	YSR Int
S3 FPC1	11	-18, 41	NIH Card
S3 FPC1	0.94	-19, 20	NIH Flanker
S3 FPC1	-27	-58, 4.0	NIH List
S3 FPC1	-19	-60, 21	NIH Pattern
S3 FPC2			
S3 FPC2	-3	-14, 8.2	MFQ SR
S3 FPC2	-5.3	-21, 10	SCARED SR
S3 FPC2	-0.3	-12, 11	YSR Ext
S3 FPC2	-0.6	-12, 11	YSR Int
S3 FPC2	3	-19, 25	NIH Card
S3 FPC2	3	-11, 17	NIH Flanker
S3 FPC2	-4	-26, 18	NIH List
S3 FPC2	-21	-51, 8.2	NIH Pattern
S3 FPC3			
S3 FPC3	13	-0.76, 27	MFQ SR
S3 FPC3	5.9	-13, 24	SCARED SR
S3 FPC3	17	3.9, 31	YSR Ext
S3 FPC3	24	9.5, 38	YSR Int
S3 FPC3	-19	-46, 7.8	NIH Card
S3 FPC3	-12	-30, 5.0	NIH Flanker
S3 FPC3	-0.71	-28, 27	NIH List
S3 FPC3	-3	-39, 34	NIH Pattern
S3 FPC4			
S3 FPC4	16	-1.3, 34	MFQ SR
S3 FPC4	25	0.22, 50	SCARED SR
S3 FPC4	17	-1.1, 35	YSR Ext
S3 FPC4	8.8	-10, 27	YSR Int
S3 FPC4	11	-25, 46	NIH Card
S3 FPC4	24	1.8, 48	NIH Flanker
S3 FPC4	-2.1	-38, 34	NIH List
S3 FPC4	-40	-88, 8.1	NIH Pattern
S3 FPC5			
S3 FPC5	-1.8	-37, 33	MFQ SR
S3 FPC5	12	-36, 60	SCARED SR
S3 FPC5	2	-34, 37	YSR Ext
S3 FPC5	-0.29	-37, 36	YSR Int
S3 FPC5	2.1	-68, 70	NIH Card

S3 FPC5	-11	-57, 33	NIH Flanker
S3 FPC5	28	-42, 99	NIH List
S3 FPC5	89	-3.6, 182	NIH Pattern

Supplementary Table 4: EHQ = Edinburgh Handedness Questionnaire; S3 FPC1 = Functional principal component 1 for state 3; MFQ SR = Mood and Feelings Questionnaire, Self Report; SCARED SR = Screen for Child Anxiety Related Disorders, Self Report; YSR Ext = Youth Self Report, Externalising Scale; YSR Int = Youth Self Report, Internalising Scale; NIH = National Institutes of Health Toolbox Cognitive function tasks; NIH Card = Card Sorting task measuring executive function; NIH Flanker = Flanker task measuring executive function and attention; NIH List = List sorting task measuring working memory; NIH Pattern = Pattern comparison task measuring processing speed.

1.9 Full Bayesian Regression Model Table for State 4

Supplementary Table 5: Regression coefficients and 95% credible intervals from Bayesian regression model for state 2.

Independent Variable	Regression Coefficient	95% Credible Interval	Dependent Variable
Intercept			
Intercept	6.5	-44, 57	MFQ SR
Intercept	-19	-91, 54	SCARED SR
Intercept	70	15, 125	YSR Ext
Intercept	33	-11, 76	YSR Int
Intercept	34	-80, 149	NIH Card
Intercept	91	34, 147	NIH Flanker
Intercept	0.94	-101, 103	NIH List
Intercept	-22	-113, 69	NIH Pattern
Sex = Female			
Sex = Female	12	4.6, 18	MFQ SR
Sex = Female	23	13, 33	SCARED SR
Sex = Female	0.08	-7.5, 7.8	YSR Ext
Sex = Female	8.8	2.8, 15	YSR Int
Sex = Female	12	-3.7, 28	NIH Card
Sex = Female	0.06	-7.7, 7.9	NIH Flanker
Sex = Female	1.5	-12, 16	NIH List
Sex = Female	11	-1.6, 24	NIH Pattern
Age			
Age	2.2	-0.66, 5.0	MFQ SR
Age	2.8	-1.3, 7.0	SCARED SR
Age	1.7	-1.4, 4.9	YSR Ext
Age	1.7	-0.77, 4.2	YSR Int
Age	0.76	-5.7, 7.3	NIH Card
Age	-1.8	-5.0, 1.4	NIH Flanker
Age	1.6	-4.0, 7.3	NIH List
Age	-1.2	-6.4, 3.9	NIH Pattern
EHQ			
EHQ	-0.05	-0.10, 0.00	MFQ SR
EHQ	-0.09	-0.16, -0.01	SCARED SR
EHQ	-0.03	-0.09, 0.02	YSR Ext
EHQ	-0.06	-0.10, -0.01	YSR Int
EHQ	0.04	-0.08, 0.16	NIH Card
EHQ	0.04	-0.01, 0.10	NIH Flanker
EHQ	0.1	0.00, 0.21	NIH List
EHQ	0.1	0.01, 0.19	NIH Pattern
N. States			
N. States	-7.6	-17, 1.7	MFQ SR
N. States	-6	-20, 7.3	SCARED SR
N. States	-11	-21, -0.31	YSR Ext
N. States	-8.7	-17, -0.52	YSR Int
N. States	9.5	-12, 31	NIH Card
N. States	4.3	-6.1, 15	NIH Flanker
N. States	10	-8.5, 29	NIH List
N. States	23	6.2, 40	NIH Pattern
N. Transitions			
N. Transitions	0.17	-3.2, 3.5	MFQ SR
N. Transitions	1.6	-3.4, 6.5	SCARED SR
N. Transitions	1.1	-2.7, 4.9	YSR Ext

N. Transitions	3.4	0.47, 6.4	YSR Int
N. Transitions	0.28	-7.6, 8.1	NIH Card
N. Transitions	1.6	-2.2, 5.5	NIH Flanker
N. Transitions	0.39	-6.5, 7.3	NIH List
N. Transitions	-0.82	-7.0, 5.2	NIH Pattern
Dominant State %			
Dominant State %	-11	-46, 24	MFQ SR
Dominant State %	12	-38, 61	SCARED SR
Dominant State %	-28	-67, 11	YSR Ext
Dominant State %	15	-16, 45	YSR Int
Dominant State %	39	-44, 121	NIH Card
Dominant State %	9.7	-30, 50	NIH Flanker
Dominant State %	64	-8.6, 136	NIH List
Dominant State %	95	30, 159	NIH Pattern
S4 FPC1			
S4 FPC1	-2.2	-14, 10	MFQ SR
S4 FPC1	1.5	-17, 19	SCARED SR
S4 FPC1	4.3	-9.4, 18	YSR Ext
S4 FPC1	1.4	-9.3, 12	YSR Int
S4 FPC1	11	-16, 39	NIH Card
S4 FPC1	8.6	-5.3, 23	NIH Flanker
S4 FPC1	-3.1	-28, 22	NIH List
S4 FPC1	10	-12, 33	NIH Pattern
S4 FPC2			
S4 FPC2	11	-1.7, 24	MFQ SR
S4 FPC2	4.2	-14, 22	SCARED SR
S4 FPC2	4.5	-9.6, 19	YSR Ext
S4 FPC2	4.4	-6.6, 15	YSR Int
S4 FPC2	-19	-48, 9.7	NIH Card
S4 FPC2	-18	-33, -3.6	NIH Flanker
S4 FPC2	-0.63	-27, 25	NIH List
S4 FPC2	22	-1.7, 45	NIH Pattern
S4 FPC3			
S4 FPC3	22	8.8, 36	MFQ SR
S4 FPC3	23	3.4, 42	SCARED SR
S4 FPC3	12	-3.3, 26	YSR Ext
S4 FPC3	22	10, 33	YSR Int
S4 FPC3	2.3	-28, 34	NIH Card
S4 FPC3	-5.6	-21, 9.7	NIH Flanker
S4 FPC3	-4.6	-31, 22	NIH List
S4 FPC3	-1.6	-26, 22	NIH Pattern
S4 FPC4			
S4 FPC4	-13	-31, 5.3	MFQ SR
S4 FPC4	-23	-49, 3.8	SCARED SR
S4 FPC4	-2.1	-22, 18	YSR Ext
S4 FPC4	-14	-30, 2.0	YSR Int
S4 FPC4	-6	-48, 36	NIH Card
S4 FPC4	6.3	-15, 27	NIH Flanker
S4 FPC4	11	-26, 48	NIH List
S4 FPC4	-29	-62, 4.5	NIH Pattern
S4 FPC5			
S4 FPC5	23	-9.9, 57	MFQ SR
S4 FPC5	78	29, 127	SCARED SR
S4 FPC5	15	-22, 53	YSR Ext
S4 FPC5	41	12, 71	YSR Int
S4 FPC5	26	-50, 102	NIH Card

S4 FPC5	11	-27, 49	NIH Flanker
S4 FPC5	-28	-96, 39	NIH List
S4 FPC5	27	-33, 88	NIH Pattern

Supplementary Table 5: EHQ = Edinburgh Handedness Questionnaire; S4 FPC1 = Functional principal component 1 for state 4; MFQ SR = Mood and Feelings Questionnaire, Self Report; SCARED SR = Screen for Child Anxiety Related Disorders, Self Report; YSR Ext = Youth Self Report, Externalising Scale; YSR Int = Youth Self Report, Internalising Scale; NIH = National Institutes of Health Toolbox Cognitive function tasks; NIH Card = Card Sorting task measuring executive function; NIH Flanker = Flanker task measuring executive function and attention; NIH List = List sorting task measuring working memory; NIH Pattern = Pattern comparison task measuring processing speed.
