# Supplementary material for: Functional analysis within latent states: A novel framework for analysing functional time series data
Source: PLoS One. 2025 Jun 27;20(6):e0326598. doi: 10.1371/journal.pone.0326598 (PMC12204692; doi:10.1371/journal.pone.0326598)
Supplement: Attachment — Submitted filename: Review_PLOS.pdf [file pone.0326598.s003.pdf]

Review of the Manuscript:

"Functional analysis within latent states: A novel framework for analysing functional time series data"

In this revised manuscript, authors propose a novel methodological framework, **FunctionaL Analysis Within LatEnt StateS** (*flawless*), for the analysis of **functional time series (FTS)** with an emphasis on **dimension reduction and latent state modeling**. Their approach integrates a **functional hidden Markov model (FHMM)** to capture the temporal dependencies and transitions between latent states within FTS, while employing **functional principal component analysis (FPCA)** to perform dimension reduction within each inferred state. This nested structure enables a more nuanced exploration of functional variation across time while maintaining interpretability. The authors demonstrate the utility of the framework using **electroencephalography (EEG) power spectral density data** from the **Healthy Brain Network study**, identifying four functional latent states associated with differences in **psychopathology and cognitive function**. By leveraging **FHMM for state modeling** and **FPCA for dimensionality reduction**, the proposed framework provides a flexible and interpretable alternative to traditional multivariate approaches for analyzing high-dimensional functional data.

Main Comment

One of the main concerns I share with a reviewer of the original submission is that the manuscript would benefit from a more comprehensive discussion of how the *flawless* framework relates to existing **FTS methodologies**. While

the proposed approach of combining **FHMM for dependency modeling** and **FPCA for dimension reduction** is interesting, there are alternative **dimension reduction techniques** specifically designed for FTS that the authors may want to acknowledge. I recommend that the authors refer to the recent review article *"A journey from univariate to multivariate functional time series: A comprehensive review"* (link), which provides a thorough overview of modern approaches in this area. In particular, **Functional Singular Spectrum Analysis (FSSA)** (see: link) offers a direct method for **dimension reduction** of FTS, in contrast to the **hybrid FHMM + FPCA approach** used in this paper. While I do not suggest additional empirical comparisons, incorporating a discussion of **FSSA and other FDA-based methods**—either in the **Introduction** or **Discussion**—would **help contextualize the proposed framework** within the broader literature and strengthen the manuscript's contributions.

Minor Comments

1. **Consistency in Writing:**

    - Ensure uniformity in equation references: e.g., use **"(1)"** and **"(2)"** instead of "Eq 1" (line 375) and "Equation 2" (line 459).
    - Standardize the notation for the number of subjects: lowercase **"n"** and uppercase **"N"** are used inconsistently (e.g., line 583 vs. Table 2).
    - Maintain consistency in referencing states: e.g., **"state 1"** (line 583) vs. **"State 1"** (line 803). Check for consistency across the manuscript.

2. **Grammar and Formatting Issues:**

    - Correct capitalization errors, such as **"dimensionality"** starting with a lowercase letter in line 98.
    - Move reference [**75**] to the end of the sentence in line 272 for better readability.
    - Avoid word repetition: e.g., "In the Results we present **results**" (line 182).

3. **Typos:**
   - Correct **"¿70%"** in line 477.
   - Fix the typo in line 519: **"sectionpresents"** → **"section presents"**.
   - Adjust the quotation mark in line 474: **'elbow'** instead of **'elbow'**.

4. **Abbreviations:**
   - Introduce abbreviations **consistently** upon first use:
     - **"FOOOF"** is first used in line 42 but only introduced in line 212. Also, there are inconsistencies in its full name: **"fitting oscillations and one over f"** (line 212) vs. **"fitting oscillations and one-over-f"** (line 335).
     - **"FHMM"** is introduced in line 72 but later written in full as **"functional Hidden Markov Model"** in multiple locations (e.g., lines 197, 213, 338, 412, 732, and Fig. 2 caption).
     - The abbreviation **"FPC"** appears multiple times (e.g., line 625) without prior introduction.

5. **Figures:**
   - In **Figure 3**, introduce **gaps between bars** in the bar chart to avoid confusion with a histogram.

6. **Review for Missed Revisions:**
   - Ensure all prior corrections were applied consistently:
     - In **line 612**, replace **"sections 3.2 and 3.3"** with their **actual section titles**.
     - In the **references section**, acronyms should be consistent—e.g., "EEG" was corrected, but **"PCA"** in reference **#10** is still written as **"Pca"**.
