# Supplementary material for: Functional analysis within latent states: A novel framework for analysing functional time series data
Source: PLoS One. 2025 Jun 27;20(6):e0326598. doi: 10.1371/journal.pone.0326598 (PMC12204692; doi:10.1371/journal.pone.0326598)
Supplement: Attachment — Submitted filename: Response to Reviewers.docx [file pone.0326598.s002.docx]

Dear Professor Yu Zhou,

Thank you for your positive decision on our manuscript. Please find our revised version attached. We believe that the manuscript is much improved and we are grateful to you and the reviewers for providing such useful feedback. We also thank both reviewers for carefully reading the manuscript and their constructive feedback.

Please find below a detailed response to your comments. These reflect the amendments in the revised manuscript.

Kind regards,
Owen Forbes and colleagues

Dear Dr. Forbes,

Thank you for submitting your manuscript to PLOS ONE. After careful consideration, we feel that it has merit but does not fully meet PLOS ONE’s publication criteria as it currently stands. Therefore, we invite you to submit a revised version of the manuscript that addresses the points raised during the review process.

Please submit your revised manuscript by Oct 06 2024 11:59PM. If you will need more time than this to complete your revisions, please reply to this message or contact the journal office at [plosone@plos.org](mailto:plosone@plos.org). When you're ready to submit your revision, log on to [https://www.editorialmanager.com/pone/](https://urldefense.com/v3/__https:/www.editorialmanager.com/pone/__;!!NVzLfOphnbDXSw!EUNjwWi0-9k3UvjYL2buNuQGph8IIiAhLLDVFxpadH2ijiSFq0Yf4xpJG1E4pXy9PexcRiilFIYJX-dUyDka2apvXw$) and select the 'Submissions Needing Revision' folder to locate your manuscript file.

Please include the following items when submitting your revised manuscript:

- A rebuttal letter that responds to each point raised by the academic editor and reviewer(s). You should upload this letter as a separate file labeled 'Response to Reviewers'.
- A marked-up copy of your manuscript that highlights changes made to the original version. You should upload this as a separate file labeled 'Revised Manuscript with Track Changes'.
- An unmarked version of your revised paper without tracked changes. You should upload this as a separate file labeled 'Manuscript'.

If you would like to make changes to your financial disclosure, please include your updated statement in your cover letter. Guidelines for resubmitting your figure files are available below the reviewer comments at the end of this letter.

If applicable, we recommend that you deposit your laboratory protocols in protocols.io to enhance the reproducibility of your results. Protocols.io assigns your protocol its own identifier (DOI) so that it can be cited independently in the future. For instructions see: [https://journals.plos.org/plosone/s/submission-guidelines#loc-laboratory-protocols](https://urldefense.com/v3/__https:/c05y1x9s.r.us-east-2.awstrack.me/L0/https:*2F*2Fjournals.plos.org*2Fplosone*2Fs*2Fsubmission-guidelines*23loc-laboratory-protocols/1/010f01917b4878d0-44f57768-890c-450d-bd42-dec78fe802d8-000000/MORHG7vArhC8YdfFOBZ3hx5huW4=173__;JSUlJSUl!!NVzLfOphnbDXSw!EUNjwWi0-9k3UvjYL2buNuQGph8IIiAhLLDVFxpadH2ijiSFq0Yf4xpJG1E4pXy9PexcRiilFIYJX-dUyDnk2xgU6w$). Additionally, PLOS ONE offers an option for publishing peer-reviewed Lab Protocol articles, which describe protocols hosted on protocols.io. Read more information on sharing protocols at [https://plos.org/protocols?utm_medium=editorial-email&utm_source=authorletters&utm_campaign=protocols](https://urldefense.com/v3/__https:/c05y1x9s.r.us-east-2.awstrack.me/L0/https:*2F*2Fplos.org*2Fprotocols*3Futm_medium=editorial-email*26utm_source=authorletters*26utm_campaign=protocols/1/010f01917b4878d0-44f57768-890c-450d-bd42-dec78fe802d8-000000/p4zfvIcVJu5mesYzAjKTCAC68po=173__;JSUlJSUl!!NVzLfOphnbDXSw!EUNjwWi0-9k3UvjYL2buNuQGph8IIiAhLLDVFxpadH2ijiSFq0Yf4xpJG1E4pXy9PexcRiilFIYJX-dUyDlw_UT8RQ$).

We look forward to receiving your revised manuscript.

Kind regards,

Yu Zhou
Academic Editor
PLOS ONE

Journal Requirements:

When submitting your revision, we need you to address these additional requirements.

**Editor’s comments**

1. Please ensure that your manuscript meets PLOS ONE's style requirements, including those for file naming. The PLOS ONE style templates can be found at 
[https://journals.plos.org/plosone/s/file?id=wjVg/PLOSOne_formatting_sample_main_body.pdf](https://urldefense.com/v3/__https:/journals.plos.org/plosone/s/file?id=wjVg*PLOSOne_formatting_sample_main_body.pdf__;Lw!!NVzLfOphnbDXSw!EUNjwWi0-9k3UvjYL2buNuQGph8IIiAhLLDVFxpadH2ijiSFq0Yf4xpJG1E4pXy9PexcRiilFIYJX-dUyDmKsdXSZg$) and 
[https://journals.plos.org/plosone/s/file?id=ba62/PLOSOne_formatting_sample_title_authors_affiliations.pdf](https://urldefense.com/v3/__https:/journals.plos.org/plosone/s/file?id=ba62*PLOSOne_formatting_sample_title_authors_affiliations.pdf__;Lw!!NVzLfOphnbDXSw!EUNjwWi0-9k3UvjYL2buNuQGph8IIiAhLLDVFxpadH2ijiSFq0Yf4xpJG1E4pXy9PexcRiilFIYJX-dUyDl4VjFrNw$)

Thank you, we have revised our manuscript according to PLOS ONE style templates.

2. **Please note that PLOS ONE has specific guidelines on code sharing for submissions in which author-generated code underpins the findings in the manuscript. In these cases, we expect all author-generated code to be made available without restrictions upon publication of the work. Please review our guidelines at** [**https://journals.plos.org/plosone/s/materials-and-software-sharing#loc-sharing-code**](https://urldefense.com/v3/__https:/journals.plos.org/plosone/s/materials-and-software-sharing*loc-sharing-code__;Iw!!NVzLfOphnbDXSw!EUNjwWi0-9k3UvjYL2buNuQGph8IIiAhLLDVFxpadH2ijiSFq0Yf4xpJG1E4pXy9PexcRiilFIYJX-dUyDmQ3IsHQw$) **and ensure that your code is shared in a way that follows best practice and facilitates reproducibility and reuse.**

- Share their code via a repository that issues persistent identifiers, such as DOIs.
- Clearly license their code using an open source license.
- Share clear documentation alongside the code that details any information needed to run the code, for example dependencies.
- State how the code can be accessed in the Data Availability Statement of their manuscript.

Thank you, we have made these changes to the code and documentation, and it is now available on Zenodo at <https://doi.org/10.5281/zenodo.14533222>. We have also updated the Data Availability Statement accordingly:

All code written in support of this manuscript is available at https://doi.org/10.5281/zenodo.14533222. All data used for this work are available through the Child Mind Institute’s Healthy Brain Network Scientific Data Portal, accessible at https://fcon_1000.projects.nitrc.org/indi/cmi_healthy_brain_network/.

**3. Thank you for stating the following financial disclosure: 
"Full list of funders: Australian Research Council Centre of Excellence for Mathematical and Statistical Frontiers, CE140100049, KM Statistical Society of Australia, PhD Scholarship, OF Queensland University of Technology, PhD Scholarship, OF International Biometrics Society, PhD Scholarship, OF"**
**Please state what role the funders took in the study.  If the funders had no role, please state: ""The funders had no role in study design, data collection and analysis, decision to publish, or preparation of the manuscript."" 
If this statement is not correct you must amend it as needed. 
Please include this amended Role of Funder statement in your cover letter; we will change the online submission form on your behalf.**

Thank you. We have added to the cover letter:
"The funders had no role in study design, data collection and analysis, decision to publish, or preparation of the manuscript."

**4. Please ensure that you refer to Figure 6 in your text as, if accepted, production will need this reference to link the reader to the figure.**

Thank you. Fig 6 is referenced at line 624, page 30:
“Eigenfunctions for state 1 plotted in Fig 6.”

**5. Please include captions for your Supporting Information files at the end of your manuscript, and update any in-text citations to match accordingly. Please see our Supporting Information guidelines for more information:** [**http://journals.plos.org/plosone/s/supporting-information**](https://urldefense.com/v3/__http:/journals.plos.org/plosone/s/supporting-information__;!!NVzLfOphnbDXSw!EUNjwWi0-9k3UvjYL2buNuQGph8IIiAhLLDVFxpadH2ijiSFq0Yf4xpJG1E4pXy9PexcRiilFIYJX-dUyDloCbFxoQ$)**.**

Thank you. We have updated our Supporting Information captions and in-text citations. Please see the revised manuscript for details.

**6. Please update your submission to use the PLOS LaTeX template. The template and more information on our requirements for LaTeX submissions can be found at** [**http://journals.plos.org/plosone/s/latex**](https://urldefense.com/v3/__http:/journals.plos.org/plosone/s/latex__;!!NVzLfOphnbDXSw!EUNjwWi0-9k3UvjYL2buNuQGph8IIiAhLLDVFxpadH2ijiSFq0Yf4xpJG1E4pXy9PexcRiilFIYJX-dUyDlp189TyA$)**.**

Thank you. We have updated to use the PLOS LaTeX template.

**Reviewer #1: PONE-D-24-19155 Review**

Functional analysis within latent states: A novel framework for analysing functional time series data

**The authors propose a nested approach to analyse EEG signals from a single electrode, Cz, using functional data analysis versions of HMM and PCA, in a large dataset, N = 503, of early adolescents aged 9–15. The paper is well written, although sometimes repetitive and with occasional claims without substantiation. Although I question the wisdom of naming a method flawless, the approach is methodologically sound and should find its way eventually into mainstream neuroimaging. However, there are several points which need to be addressed before accepting the paper, and some recommendations that would make the paper and method more attractive to the neuroimaging community.**

Thank you for your thoughtful and constructive feedback on our manuscript. We appreciate the time and effort you have put into reviewing our work. We have carefully considered your comments and have made several revisions to improve the paper. Below, we address each of your points in turn.

**Comment 1 - major
The authors describe the results but provide no interpretation with respect to neuroscience. How, for example, do these findings align or not with EEG results from other studies with children (e.g. ADHD) or adults (HMM analysis)? How are the canonical frequency bands related to psychopathy and cognition, and do the findings in this paper agree or challenge previous findings? Without some interpretation of the results, the paper may end up being considered interesting but irrelevant for many neuroscience researchers who are faced with a multitude of different methods to apply to their data.**

Response: Thank you for your comment. In the Discussion section, we have added the following paragraph to the analysis to discuss an example of how our research compares to previous findings relating EEG power bands and cognitive function/psychopathology, and how our method provides deeper insights.

“Our findings extend on previous research on EEG characteristics in neurodevelopmental conditions and psychopathology. For instance, the high delta and low alpha power observed in our State 1, associated with poorer cognitive function and high psychopathology, aligns with previous studies linking higher delta-alpha ratios to increased incidence of social anxiety in adolescents [73] and autism spectrum disorder in children [70], as well as poorer cognitive function following brain injury or stroke [71,72]. However, our functional approach reveals more nuanced relationships between frequency characteristics and cognitive measures. For instance, the association we found between scores on FPC4 in State 1 and poorer cognitive function suggests that the interplay between theta, low-frequency alpha and beta power, indicating the presence of lower peak frequencies for alpha and beta activity, may be more informative than examining these bands in isolation, as is common in traditional EEG analyses. This highlights the potential of our functional approach to uncover more complex spectral signatures of cognitive function and psychopathology, going beyond simple ratios to consider the entire spectral profile and its dynamics over time.”

**Comment 2 - major
The authors choose to analyse the EEG signals from Cz (supplementary motor area and sensorimotor cortex, motor coordination, planning and execution) but regress the results against psychopathology and cognitive measures, the latter which are associated with prefrontal and frontal cortex. Re-running the analysis with Fp1/Fp2 would provide a higher level of credibility of the results, or alternativey, providing an explanation why Cz would capture signals related to prefrontal/frontal activity.**

Thank you for your comment. We acknowledge the utility of analysing data from Fp1/Fp2 electrodes given their location over prefrontal regions that potentially have more direct relevance to cognitive function and psychopathology. However, we chose to maintain our focus on Cz data for several key reasons:

1. Signal quality and consistency: The Cz electrode typically provides high-quality signals with reduced susceptibility to artifacts compared to frontal electrodes, which are more affected by eye movements and muscle activity. This was particularly important given our focus on detailed spectral analyses.
2. Implementation considerations: Our goal was to develop methods that could be readily implemented across clinical and research settings, using a variety of EEG headsets and acquisition setups. The consistent placement and typically good signal quality of Cz supports this translational objective.
3. Methodological focus: The primary aim of this paper was to introduce and validate the ‘flawless’ analysis framework. While analysing Fp1/Fp2 data could provide valuable insights, we believe the current analyses using Cz data sufficiently demonstrate the utility of our methodological approach.

We have addressed this limitation in the manuscript by adding explanatory text regarding our use of Cz and acknowledging the value of extending analyses to frontal electrodes in future work. For this application we believe that the analyses using Cz are sufficient, and have included this explanation in the manuscript as suggested. We have added the following sentences to the Methods and updated the Limitations to address our use of data from the Cz electrode:

Methods:
“We focused specifically on the Cz electrode, which is often used as a reference electrode due to its central location and typically high-quality signal [74]. Cz was used as the reference electrode in the HBN EEG acquisition protocol. This placement provides a good balance between signal quality and reduced susceptibility to artifacts, and the consistent placement of Cz [75] could support implementation across various clinical and research settings.”

Limitations:
“While our focus on the Cz electrode provided valuable insights, we acknowledge that this single-electrode approach limits our ability to capture spatial patterns of brain activity... Another avenue for future extension could include analysis of data from multiple EEG channels, capturing activity from different brain regions such as prefrontal and frontal activity, which may be more directly related to some of the cognitive and psychopathological measures we studied.”


**Comment 3 - minor
Abstract - The flawless framework offers superior flexibility and nuance. This is unsubstantiated and unnecessary.**

Comment 3: We have removed this statement from the Abstract and instead focused on describing the key features of the framework and the unique aspects of our work relative to specific aspects of other analytical approaches in the field.

**Comment 4 – minor
Page 3
Ref 11 – date is missing in the reference.
Ref 15 is a preprint and does not represent ‘common practice’ in EEG research.**

Comment 4: Thank you for pointing out these reference issues. We have updated Reference 11 to include the missing date. Regarding the use of standardised frequency bands in EEG research, we have replaced Reference 15 with a more established source. Specifically, we now cite Newson & Thiagarajan (2019), a comprehensive review that demonstrates the continued widespread use of canonical frequency bands (delta, theta, alpha, beta, gamma) in contemporary EEG studies across various psychiatric disorders.

**Comment 5 – minor
Page 4
HMM … latent states – remove ‘dominant modes’ as these are relevant for FPCA.**

Comment 5: We have removed "dominant modes" from the description of HMM and latent states, as you correctly pointed out that this is more relevant to FPCA.

**Comment 6 – minor
Page 5 and others
The use of ‘longitudinal’ is incorrect. The method can be applied to any time-varying functional data.**

Comment 6: We appreciate your observation about the incorrect use of "longitudinal." We have revised this labelling throughout the manuscript to clarify that the method can be applied to any time-varying functional data.

**Comment 7 – minor
Page 5
‘In this work… using regression of clustering approaches’. There is no methodology outlined in the paper for regression of clustering approaches.**

Comment 7: Thank you for your comment – we have removed this sentence as suggested.

**Comment 8 – minor
Page 6 – repetition – remove ‘building a biobank’**

Comment 8: We have removed the repetition of "building a biobank" as suggested.

**Comment 9 – minor
Page 7 and page 32
‘predominantly allocated to each latent state’. ‘…mainly… This implies that individuals were allocated to multiple dominant states which I do not believe was the case.**

Comment 9: Thank you for your feedback. We have clarified this by changing the description to “with 80% or more of their time allocated to one latent state”

**Comment 10 – minor
Page 10 ‘clinical relevance’ – I believe this should be clinical utility i.e. it is easy to measure in the clinic.
‘choosing high quality measures’ – unsubstantiated.**

Comment 10: We have changed "clinical relevance" to "clinical utility" to better reflect the ease of measurement in clinical settings. We have also removed the description of “high quality” measures.

**Comment 11 – minor
Page 13 – Repetition – suggest removing ‘Differing from a …’**

Comment 11: Thank you for pointing this out. We have removed this sentence to reduce repetition and improve the flow of the text.

**Comment 12 – minor
Page 14 – ‘Visual identification’ and ‘averaged’. The method would be more robust if you could find an algorithmic way to distinguish the centroids. What are the implications of averaging the centroids for initiation?**

Comment 12: We appreciate your suggestion for improving the robustness of our method. We have added to the Limitations section a brief discussion of the implications of our current approach and potential future improvements:

“While our current approach for initialising the FHMM algorithm relies on visual identification and averaging of stable centroids, we acknowledge that an algorithmic method for distinguishing centroids could enhance the robustness of our approach. The implications of averaging centroids for initialisation include potential loss of some fine-grained distinctions between states, but in this case it helps to identify stable, consistent patterns across subsamples and improve the stability of algorithm performance. Future work could explore algorithmic methods for initial centroid identification, such as a functional implementation of k-means++ [51], to further improve the reliability and reproducibility of our results.”

**Comment 13 – minor
Page 15 – What criteria did you use to ‘assess’ the scree plots? Also ‘on inspection’ – for what?**

Comment 13: We have clarified our criteria for assessing scree plots and what we were inspecting. The revised text now reads:

" To select functional PCs to retain, we assessed scree plots by looking for the 'elbow' point where the rate of decrease in explained variance begins to level off. On inspection of these plots, we looked for the number of components that cumulatively explained a substantial proportion (>70\%) of the total variance while balancing parsimony.”

**Comment 14 – minor
Page 16 and elsewhere – Reference to section 3.1 etc. which do not exist.**

Comment 14: Thank you, we have removed references to these section numbers.

**Comment 15 – minor
Page 17 – Table 1 EHQ is not defined, and the order of the abbreviations does not follow the order in the table.
‘FOOOF performance….’ What is this and why is it here?**

Response: Thank you for identifying these issues. We have made the following changes:

1. We have defined EHQ (Edinburgh Handedness Questionnaire) in the table footnote.
2. We have reordered the abbreviations in the table footnote to match the order in the table.
3. FOOOF performance metrics relate to fit / diagnostic measures of the “`fitting oscillations and one over f` (FOOOF) algorithm” (Donoghue et al., 2020). For clarity, we have included the full phrase and the acronym together, along with repeating the corresponding citation here.

**Comment 16 – minor
Page 18 and elsewhere – Figure 2 in the text should be Figure 3, etc for all subsequent figures.**
Comment 16: Thank you. We have reviewed and corrected all figure references throughout the manuscript to ensure they accurately correspond to the correct figure numbers.

**Comment 18 – major
Page 19 – Section ‘Comparing Psychopathology….’ And Figure 5. I would like to see ANOVAs on the psycho and cognitive measures across the 4 dominant state groups. The statements on contrasts among the groups is not statistically substantiated.**

Comment 18: Thank you for this suggestion. We would like to clarify that this section presents an exploratory descriptive characterisation intended to illustrate patterns in the data, rather than make inferential claims about statistical significance or broader generalisability of the differences between groups as would available through ANOVA analyses. While interesting, such analysis is beyond the scope of this (already lengthy) paper. The aim was to describe the characteristics of individuals who spent substantial time in each state, to provide context for the subsequent detailed analyses using Bayesian regression models. We have added text to the manuscript clarifying the descriptive nature of this analysis, without implying any generalisable inferences regarding statistically significant differences between dominant states:

“This descriptive analysis aims to characterise patterns in psychopathology and cognitive function between individuals who spent substantial time in each state, providing context for subsequent detailed analyses, rather than make inferential claims about statistical significance or broader generalisability of these differences.”

**Comment 19 – minor
Page 23 and elsewhere - ‘individuals with higher scores...’ What scores – do the authors mean the FPCA weightings?**

Comment 19: Thank you for pointing out this ambiguity. We have clarified throughout the manuscript that we are referring to FPCA scores. For example, we have revised the text to read "individuals with higher scores on this functional principal component" to make it clear we are discussing FPCA scores.

**Comment 20 – minor
Page 26-31 – I would suggest putting Table 3 and 4 together so that readers can compare the regression results across State1 and State 3 dominance groups.**

Comment 20: Thank you for this suggestion to improve the presentation of our results. We appreciate the intent to facilitate easier comparison across State 1 and State 3 dominance groups. However, we have decided to keep Tables 3 and 4 separate to emphasise that these results come from distinct models for each state. This separation helps maintain clarity about the independence of these analyses and prevents potential misinterpretation of the results as coming from a single model. To aid in comparison, we have ensured that both tables follow the same format and order of variables. Additionally, we have added a note in the text directing readers to compare across these tables for insights into differences between State 1 and State 3 dominance groups:

“We encourage interested readers to compare these Bayesian regression results across Tables 3 and 4.”


**Comment 21 – major
Page 26 – I would suggest putting a brief summary of the results at the start of the section ‘Bayesian Regression…’ highlighting that FHMM dynamics were statistically associated with NIH Flanker in the State 1 group, and YSR Ext, YSR Int, SCARED SR, and NIH Pattern in the State 3 group. With the additional FPCA analysis, individual weightings were statistically associated with NIH Pattern, NIH Card, NIH Flanker and NIH List in the State 1 group, and weightings in the State 3 group were statistically associated with YSR Ext, YSR Int, SCARED SR, and NIH Flanker.
These findings and the insights they provide should be discussed in the discussion section.**

Comment 21: Thank you for this helpful suggestion. We have added the following summary paragraph at the start of that section:
“The Bayesian regression models revealed several substantial associations between functional analysis outputs and health measures. For state 1, FHMM dynamics showed the number of transitions was positively associated with NIH Flanker performance. FPCA weightings in state 1 showed negative associations between FPC3 and NIH Pattern performance, and between FPC4 and performance on NIH Card, Flanker and List tasks. For state 3, FHMM dynamics showed the number of states was positively associated with YSR Externalising, while number of transitions was negatively associated with YSR Internalising. Time spent in state 3 was negatively associated with SCARED SR and positively associated with NIH Pattern performance. FPCA weightings in state 3 showed positive associations between FPC3 and both YSR scales (Externalising and Internalising), and between FPC4 and both SCARED SR and NIH Flanker performance.”

These effects are discussed in the following area of the Discussion:

“Using FPCA stratified by latent state, and subsequent Bayesian regression models, we have also identified associations between temporal dynamics of latent state occupancy, frequency characteristics within states, and a variety of important health outcomes. Findings that stand out from these analyses include that scores on FPC3 and FPC4 in state 1, with prominent features including power in the theta, low-frequency alpha and beta-2 ranges, were associated with poorer cognitive function relative to other individuals for whom state 1 was dominant (Table 2). For state 3, scores on FPC3 and FPC4 were associated with higher scores on several psychopathology measures, indicating that lower theta power, higher alpha power, and higher frequency beta peaks may be indicative of greater psychopathology among individuals for whom state 3 was dominant.

Our regression models also revealed a number of contrasting effects of state occupancy dynamics between state 1 and state 3. In state 1, number of transitions between states was associated with improved executive function, and weakly associated with improved working memory. In state 3, number of transitions was associated with reduced psychopathology across all four measures. In state 1, proportion of time spent in dominant state was weakly associated with higher psychopathology and poorer cognitive function across several measures. In state 3, proportion of time spent in the dominant state was associated with lower anxiety symptoms and internalising psychopathology, and better processing speed. These results are preliminary, and while the mechanisms are currently unclear for the differences observed here in the associations of health measures with temporal dynamics of latent state occupancy, the contrasting effects between states displays the detailed characteristics from functional analyses that differentiate individuals within and between dominant latent states.”

**Comment 22 – minor
Page 32 – repetition – remove second paragraph of the discussion.**

Comment 22: We have removed the second paragraph of the discussion as suggested to eliminate repetition and improve the flow of the text.

**Comment 23 – major
Page 33 - ‘These regresions….’ This is a description of the results which should eb interpreted in the context of previous findings in EEG.
‘…FDA methods can offer…’ – the reference to support the claim comes from 2014 – and the claim is exaggerated.**

Comment 23: Thank you for your comment. We have modified the strength of this claim:

“Functional data analysis (FDA) methods can offer different analytical perspectives compared to multivariate parametric approaches with manually extracted features, particularly in their ability to consider the complete functional form of the data.”

Regarding discussion of regression results, we have interpreted these findings in the context of previous EEG research in the following paragraphs (page 33-34):

“Our findings extend on previous research on EEG characteristics in neurodevelopmental conditions and psychopathology. For instance, the high delta and low alpha power observed in our State 1, associated with poorer cognitive function and high psychopathology, aligns with previous studies linking higher delta-alpha ratios to increased incidence of social anxiety in adolescents \citep{schmidt2022frontal} and autism spectrum disorder in children \citep{chan2007quantitative}, as well as poorer cognitive function following brain injury or stroke \citep{leon2009delta, aminov2017acute}. However, our functional approach reveals more nuanced relationships between frequency characteristics and cognitive measures. For instance, the association we found between scores on FPC4 in State 1 and poorer cognitive function suggests that the interplay between theta, low-frequency alpha and beta power, indicating the presence of lower peak frequencies for alpha and beta activity, may be more informative than examining these bands in isolation, as is common in traditional EEG analyses. This highlights the potential of our functional approach to uncover more complex spectral signatures of cognitive function and psychopathology, going beyond simple ratios to consider the entire spectral profile and its dynamics over time.

Overall these results demonstrate unique insights that are available through the \textit{flawless} analysis framework. They may be indicative of interaction effects of state occupancy dynamics which vary according to dominant state, and suggest the presence of further subgroups or phenotypes which could be identified on the basis of latent state dynamics and frequency characteristics within states. The centroids of functional latent states identified here bear a close resemblance to the average power spectral densities identified across 5 clusters in our previous work \citep{forbes2022EEG}. The next planned phase of this work will include formal replication and comparison of \textit{flawless} with the previous methodology used to identify resting state EEG phenotypes. These findings warrant further investigation, for which a possible approach could be clustering individuals based on \textit{flawless} outputs to develop more detailed data-driven phenotypes of resting state brain activity.

**Comment 24 – minor
Page 34 – ‘…decreased interpretability...’ – this is a critique of alternative methods, but the method in this paper lacks interpretation!**

Comment 24: We acknowledge this valid point. We have revised this section to focus on the unique strengths of our approach rather than critiquing others. The revised text now reads: “By combining functional methods that analyse these individual domains in a nested structure, our method enables analysis of inter-related patterns of features between the levels of latent state characteristics, temporal patterns of state occupancy, and functional frequency characteristics within states, while also generating specific insights at each of these levels of analysis.”

**Comment 25 – minor
Page 35 – ‘… well validated’. Again, this statement is unsubstantiated. What type of validation are you referring to? Where are the citations for the validation?**

Comment 25: We accept this comment and have revised the sentence to more accurately reflect the status of FPCA in the literature: “For this work we chose to use FPCA, as it is a widely used and well-established method for functional data reduction (Wang et al., 2016; Shang, 2014), making it an appropriate choice for this initial effort to combine functional latent state and data reduction methods in a nested framework.”

**Inconsistency – dimension reduction, dimensional reduction – dimensionality reduction is more commonly used in neuroscience.**

Thank you. We have updated this to “dimensionality reduction” throughout the manuscript for consistency.

**‘… significantly enhance our understanding…’ The results are descriptive – maybe contribute to the understanding otherwise the statement is an exaggeration.**

Thank you. We have updated this phrase to “contribute to our understanding”

Reviewer #2:

**The manuscript titled “Functional analysis within latent states: A novel framework for analysing functional time series data” proposes a novel method “flawless” based on functional data analysis (FDA) of time series data. The proposed framework uses functional principal component analysis (FPCA) and functional hidden Markov model (fHMM) to identify latent states in EEG signals. The authors explore the relationship between the latent states and measures of psychopathology and cognitive function provided alongwith the EEG data. The authors approach to predict the latent states and their association with behavorial measures is interesting and of wide interest to the community. However I have some reservations about the manuscript mainly pertaining to the efficacy and presentation of the method.**

Thank you for your feedback. We have responded to each of your comments below.

**In the introduction section of the manuscript, there are hardly any mention of fda based methods for exploring brain signals. Specifically, authors should include previous work in EEG signals based of FDA, FPCA and its variants. The authors should also include studies on EEG classification using HMM latent states. Finally, the literature review should also include latest methods and results using the EEG data from Healthy Brain Network the authors use in the study. The literature review section in the manuscript in general needs to be re-written.**

Thank you for your helpful suggestions to strengthen our literature review. We have added two new paragraphs to better contextualize our work within existing FDA and HMM applications to EEG analysis, and added two key references addressing HMM applications to EEG data: Kember et al. (2023), who applied HMM to HBN EEG data to study ADHD, and Shappell et al. (2021), who used Hidden semi-Markov Models to study brain state dynamics in ADHD

First, we have expanded our discussion of FDA and FPCA applications in EEG analysis, making more explicit the contributions of papers we had cited. We added the following paragraph:

"In neuroscience, functional data are common objects of investigation, with several studies demonstrating FDA's utility for analysing brain activity data varying over the functional domains of time, space and frequency \cite{hasenstab2017multi, scheffler2020hybrid, xie2019feature}. For example, Hasenstab et al. developed a multi-dimensional FPCA approach for EEG analysis, while Scheffler et al. extended this to handle region-referenced longitudinal EEG data. Xie & Lawniczak demonstrated FDA's value for spectral analysis of epileptic EEG signals. These studies show how FDA methods can effectively handle the high dimensional, complex temporal dependence structure inherent in brain activity data \cite{margaritella2021parameter}.”

Second, we have added a new paragraph highlighting recent HMM applications to EEG data, including work using the HBN dataset:

"Recent work has demonstrated the value of HMM approaches specifically for analyzing brain state dynamics in neurodevelopmental conditions. Kember et al. applied HMM to resting-state EEG data from the HBN dataset to study network properties in ADHD, identifying distinct electrophysiological states characterized by oscillatory power patterns and finding that dwelling in states with high alpha/beta power supported better response control. Similarly, Shappell et al. used Hidden semi-Markov Models to show that children with ADHD spend less time in anticorrelated network states and more time in hyperconnected states compared to typically developing children. These studies highlight how latent state modeling can reveal important differences in brain dynamics associated with neurodevelopmental conditions."

These additions better situate our work within the existing literature on FDA and HMM applications to EEG analysis, and highlight recent relevant work using the HBN dataset.

**The FDA portion of the method section is poorly written and requires more details. Given that this is primarily a method paper, the authors should elaborate on how a time series data is converted to a functional data and mention the assumptions and constraints underlying the functional data obtained from a time series. The authors should also specially mention that FDA works on only single channel and hence spatio-temporal latent states or signal analysis could not be performed with the proposed method.**

Thank you for highlighting the need for more clarity around the functional data conversion process and its limitations. We have made several changes to address these points:

1. We have added a new paragraph in the introduction explaining how EEG time series data is converted to functional form through spectral decomposition and smoothing. This includes discussion of both general smoothing approaches like B-splines and our specific use of the FOOOF algorithm for neural power spectra:

"When applying FDA to time series data like EEG recordings, the data must first be transformed into a functional form and appropriately smoothed. In this work, we convert EEG time series data into power spectral density (PSD) functions through frequency decomposition, producing curves that represent the distribution of signal power across frequencies at each time point. These PSDs must then be smoothed to create functional data objects - while various approaches like B-spline smoothing can be used for this purpose \citep{ramsay2009smoothing}, we employ the FOOOF algorithm \citep{donoghue2020parameterizing} which is specifically designed to separate periodic and aperiodic components in neural power spectra. This transformation assumes that the underlying signal can be meaningfully decomposed into frequency components, and that these components vary smoothly over the frequency domain. An important constraint of our approach is that we analyse data from a single EEG channel, which means we cannot capture spatio-temporal dynamics or conduct analyses of functional connectivity between brain regions. While multi-channel FDA approaches exist \citep{hasenstab2017multi}, we focus on single-channel analysis to maximise clinical utility and interpretability while demonstrating the core principles of our nested functional analysis framework."

2. We have clarified key assumptions about the functional data conversion process, including:

- The assumption in this setting that the signal can be meaningfully decomposed into frequency components

- The assumption of smooth variation over the frequency domain

- The requirement for appropriate smoothing to create functional data objects

- The specific benefits of using FOOOF for neural power spectra

3. We have explicitly acknowledged the single-channel limitation of our approach, noting that while multi-channel FDA approaches exist (Hasenstab et al., 2017), we deliberately focus on single-channel analysis to maximise clinical utility and interpretability. This means we cannot capture spatio-temporal dynamics or functional connectivity between brain regions.

These additions provide context for understanding the capabilities and limitations of our methodological framework, as well as the technical process of converting time series data to functional form.

**A pseudo code of the algorithm along with the framework as given in Figure 1 will also help the readers to better understand the steps involved in flawless and make it easier to implement.**

Thank you for this helpful suggestion. We have provided pseudocode of the involved steps on page 10:

**I have the most reservation on the results sections. Although the authors mention in the abstract that “compared to traditional multivariate statistical methods, the flawless framework superior flexibility and nuance”, but I find no demonstration of the author’s claim. Although the proposed algorithm looks promising, the authors failed to show its superior performance as the flawless is not compared with any other method. The authors must demonstrate that indeed flawless performance is better than traditional methods by comparing it with traditional methods and demonstrating the results. The authors could demonstrate the method’s performance when using the same algorithm but using time series data instead of functional data. Since the authors are using EEG data from a public database, they have to additionally demonstrate the superior performance of the proposed method when compared with other recent methods used on the same data. Finally, along with comparisons with traditional multivariate methods, the authors should also compare with recent FDA based methods. Overall the manuscript is interesting and proposed framework looks efficient. However the manuscript needs improvement in the literature review section in the Introduction and the results using proposed methods needs to be compared with other state-of-the-art methods.**

Thank you for this feedback about our Results section and making comparative claims. We have changed the statement about 'superior flexibility and nuance' to more precisely articulate the specific contribution of our work. Rather than comparing against traditional multivariate methods broadly, we now focus on describing the unique aspects of our nested analytical framework: specifically, how it enables analysis of both temporal dynamics through FHMM and detailed frequency characteristics through FPCA within matched latent states.

We note that the goal of this work was not to demonstrate superior performance against existing methods, but rather to introduce a novel analytical framework that combines functional analysis tools in a way that preserves interpretability at multiple levels - from latent state characteristics, to temporal dynamics, to frequency domain variation within states. As a result, there are no specifically comparable methods which we can directly benchmark performance against, since our approach has unique features and analytical goals which are not present in other analytical approaches we have seen implemented. To ameliorate this concern, we have revised the manuscript throughout the Results and Discussion to remove comparative claims and instead focus on describing these specific methodological features.

We have also removed the broad claim about 'superior flexibility and nuance' from the abstract and replaced it with more specific language about the framework's ability to provide comprehensive insights into the interplay between latent state characteristics, state occupancy dynamics, and functional attributes within states, while maintaining interpretability at each level.

The following section of the Discussion has been revised to emphasise the specific features of our method, removing unsupported comparative claims:

“…However, our functional approach reveals more detailed patterns of relationships between frequency characteristics and cognitive measures. For instance, the association we found between scores on FPC4 in State 1 and poorer cognitive function suggests that the interplay between theta, low-frequency alpha and beta power, indicating the presence of lower peak frequencies for alpha and beta activity, may be more informative than examining these bands in isolation, as is common in traditional EEG analyses. This highlights the potential of our functional approach to uncover more complex spectral signatures of cognitive function and psychopathology, going beyond simple ratios to consider the entire spectral profile and its dynamics over time.

Overall these results demonstrate unique insights that are available through the \textit{flawless} analysis framework. They may be indicative of interaction effects of state occupancy dynamics which vary according to dominant state, and suggest the presence of further subgroups or phenotypes which could be identified on the basis of latent state dynamics and frequency characteristics within states. This demonstrates the unique insights available through our nested FDA framework relative to other analytical approaches. The centroids of functional latent states identified here bear a close resemblance to the average power spectral densities identified across 5 clusters in our previous work \citep{forbes2022EEG}. The next planned phase of this work will include formal replication and comparison of \textit{flawless} with the previous methodology used to identify resting state EEG phenotypes. These findings warrant further investigation, which we intend to develop in the next stage of this work with a clustering-based approach using \textit{flawless} outputs to develop more detailed data-driven phenotypes of resting state brain activity.

Functional data analysis (FDA) methods can offer different analytical perspectives compared to multivariate parametric approaches with manually extracted features, particularly in their ability to consider the complete functional form of the data \citep{shang2014survey}. FDA is a recent area of growth in statistical research, and our work contributes a novel approach for nesting multiple FDA tools together to analyse temporal, latent state and frequency characteristics in time-varying functional data. By integrating these approaches in a nested structure, we are able to generate richer insights into functional and time-frequency characteristics of time-varying functional data than those available through using the individual methods in isolation. At the first stage, FHMM analysis provides insight into latent state characteristics and temporal dynamics of latent state occupancy. This stage also importantly enables the analysis to compare ‘like with like’ - by stratifying resting state brain activity data by allocation to latent states, we can apply FPCA to understanding the functional harmonics in the frequency domain that distinguish individuals while occupying matched latent states. Without this method, applying FPCA alone to time series of functional data over individuals would be conflating variation across multiple distinct latent states, and disregarding temporal dynamics.”

Thank you for taking the time to review our manuscript. We believe it is much improved by the changes we have made in response to your comments.
