# Supplementary material for: Functional analysis within latent states: A novel framework for analysing functional time series data
Source: PLoS One. 2025 Jun 27;20(6):e0326598. doi: 10.1371/journal.pone.0326598 (PMC12204692; doi:10.1371/journal.pone.0326598)
Supplement: Attachment — Submitted filename: Response to Reviewers_OF_2025-04-11.docx [file pone.0326598.s004.docx]

Dear Yu Zhou,

Thank you for your positive decision on our manuscript. Please find our revised version attached. We are grateful to you and the reviewers for providing useful feedback. We have addressed each of the comments below, in turn.

We believe that the manuscript is much improved – we hope that you agree, and find that the paper is ready for publication in PLOS ONE.

Kind regards,
Owen Forbes

**Reviewer Comments
In this revised manuscript, authors propose a novel methodological framework,**

**FunctionaL Analysis Within LatEnt StateS (flawless), for the**

**analysis of functional time series (FTS) with an emphasis on dimension**

**reduction and latent state modeling. Their approach integrates**

**a functional hidden Markov model (FHMM) to capture the temporal**

**dependencies and transitions between latent states within FTS, while employing**

**functional principal component analysis (FPCA) to perform**

**dimension reduction within each inferred state. This nested structure enables**

**a more nuanced exploration of functional variation across time while maintaining**

**interpretability. The authors demonstrate the utility of the framework**

**using electroencephalography (EEG) power spectral density**

**data from the Healthy Brain Network study, identifying four functional**

**latent states associated with differences in psychopathology and cognitive**

**function. By leveraging FHMM for state modeling and FPCA**

**for dimensionality reduction, the proposed framework provides a flexible**

**and interpretable alternative to traditional multivariate approaches for**

**analyzing high-dimensional functional data.**

Thank you for your valuable feedback. We have responded to each of your comments in detail below.

**Main Comment**

**One of the main concerns I share with a reviewer of the original submission is**

**that the manuscript would benefit from a more comprehensive discussion of**

**how the flawless framework relates to existing FTS methodologies. While**

**the proposed approach of combining FHMM for dependency modeling**

**and FPCA for dimension reduction is interesting, there are alternative**

**dimension reduction techniques specifically designed for FTS that the**

**authors may want to acknowledge. I recommend that the authors refer to**

**the recent review article “A journey from univariate to multivariate functional**

**time series: A comprehensive review” (link), which provides a thorough**

**overview of modern approaches in this area. In particular, Functional**

**Singular Spectrum Analysis (FSSA) (see: link) offers a direct method**

**for dimension reduction of FTS, in contrast to the hybrid FHMM +**

**FPCA approach used in this paper. While I do not suggest additional empirical**

**comparisons, incorporating a discussion of FSSA and other FDAbased**

**methods—either in the Introduction or Discussion—would help**

**contextualize the proposed framework within the broader literature and**

**strengthen the manuscript’s contributions.**

Thank you for this valuable suggestion to better contextualise our methodology within the FTS literature. We have added discussion of alternative FTS approaches, including FSSA and recent methodological advances, in both the Introduction and Discussion sections. We acknowledge the mathematical elegance and computational efficiency of direct FTS dimension reduction methods like FSSA. However, we explain how our nested approach serves different analytical goals by prioritizing interpretability through separate but connected levels of analysis. This allows us to capture both broad state-based patterns and detailed functional variation while maintaining clear interpretability at each level - a key consideration for clinical applications. The additions help position flawless as a complementary approach to existing FTS methods, with distinct benefits for applied researchers in neuroscience and other fields.

We have added the following paragraph to the Introduction from line 70:

“While various approaches exist for analysing functional time series (FTS) data, 70

including direct dimension reduction methods like Functional Singular Spectrum 71

Analysis (FSSA) [18], our goal is to develop a framework that preserves interpretability 72

across multiple levels of analysis while capturing both temporal dynamics and 73

functional characteristics. Recent advances in FTS methodology have provided 74

sophisticated tools for dimensionality reduction and forecasting in both univariate and 75

multivariate contexts [19]. However, for neuroscientific applications, maintaining clear 76

interpretability of outputs at each analytical level is crucial for clinical relevance and 77

practical utility.”

We have added the following paragraph to the Discussion from line 849:

“While various approaches exist for analysing functional time series (FTS) data, including direct dimension reduction methods like Functional Singular Spectrum Analysis (FSSA) [18], our goal is to develop a framework that preserves interpretability across multiple levels of analysis while capturing both temporal dynamics and functional characteristics. Recent advances in FTS methodology have provided sophisticated tools for dimensionality reduction and forecasting in both univariate and multivariate contexts \cite{haghbin2024journey}. However, for neuroscientific applications, maintaining clear interpretability of outputs at each analytical level is crucial for clinical relevance and practical utility.”

**Minor Comments**

**1. Consistency in Writing:**

**• Ensure uniformity in equation references: e.g., use “(1)” and**

**“(2)” instead of “Eq 1” (line 375) and “Equation 2” (line 459).**

**• Standardize the notation for the number of subjects: lowercase**

**“n” and uppercase “N” are used inconsistently (e.g., line 583 vs.**

**Table 2).**

**• Maintain consistency in referencing states: e.g., “state 1” (line**

**583) vs. “State 1” (line 803). Check for consistency across the**

**manuscript.**

Thank you for your detailed and valuable feedback. We have made these edits as suggested.

**2. Grammar and Formatting Issues:**

**• Correct capitalization errors, such as “dimensionality” starting**

**with a lowercase letter in line 98.**

**• Move reference [75] to the end of the sentence in line 272 for better**

**readability.**

**• Avoid word repetition: e.g., “In the Results we present results”**

**(line 182).**

Thank you for your feedback. We have made these edits as suggested.

**3. Typos:**

**• Correct “.70%” in line 477.**

**• Fix the typo in line 519: “sectionpresents”→“section presents”.**

**• Adjust the quotation mark in line 474: ‘elbow’ instead of ’elbow’.**

Thank you for your feedback. We have made these edits as suggested.

**4. Abbreviations:**

**• Introduce abbreviations consistently upon first use:**

**– “FOOOF” is first used in line 42 but only introduced in line**

**212. Also, there are inconsistencies in its full name: “fitting**

**oscillations and one over f” (line 212) vs. “fitting**

**oscillations and one-over-f” (line 335).**

**– “FHMM” is introduced in line 72 but later written in full as**

**“functional Hidden Markov Model” in multiple locations**

**(e.g., lines 197, 213, 338, 412, 732, and Fig. 2 caption).**

**– The abbreviation “FPC” appears multiple times (e.g., line**

**625) without prior introduction.**

Thank you for your feedback. We have made these edits as suggested to improve consistency in initial introduction and repeat usage of acronyms/abbreviations.

**5. Figures:**

**• In Figure 3, introduce gaps between bars in the bar chart to**

**avoid confusion with a histogram.**

Thank you for your feedback. We have revised Figure 3 to have gaps between the bars to avoid confusion with a histogram.

**6. Review for Missed Revisions:**

**• Ensure all prior corrections were applied consistently:**

**– In line 612, replace “sections 3.2 and 3.3” with their actual**

**section titles.**

**– In the references section, acronyms should be consistent—e.g.,**

**“EEG” was corrected, but “PCA” in reference #10 is still**

**written as “Pca”.**

Thank you for your feedback. We have made these edits as suggested.
